# Genomics of Aerobic Cellulose Utilization Systems in Actinobacteria

**DOI:** 10.1371/journal.pone.0039331

**Published:** 2012-06-18

**Authors:** Iain Anderson, Birte Abt, Athanasios Lykidis, Hans-Peter Klenk, Nikos Kyrpides, Natalia Ivanova

**Affiliations:** 1 U.S. Department of Energy Joint Genome Institute, Walnut Creek, California, United States of America; 2 Leibniz Institute DSMZ – German Collection of Microorganisms and Cell Cultures, Braunschweig, Germany; Tel Aviv University, Israel

## Abstract

Cellulose degrading enzymes have important functions in the biotechnology industry, including the production of biofuels from lignocellulosic biomass. Anaerobes including Clostridium species organize cellulases and other glycosyl hydrolases into large complexes known as cellulosomes. In contrast, aerobic actinobacteria utilize systems comprised of independently acting enzymes, often with carbohydrate binding domains. Numerous actinobacterial genomes have become available through the Genomic Encyclopedia of Bacteria and Archaea (GEBA) project. We identified putative cellulose-degrading enzymes belonging to families GH5, GH6, GH8, GH9, GH12, GH48, and GH51 in the genomes of eleven members of the actinobacteria. The eleven organisms were tested in several assays for cellulose degradation, and eight of the organisms showed evidence of cellulase activity. The three with the highest cellulase activity were Actinosynnema mirum, Cellulomonas flavigena, and Xylanimonas cellulosilytica. Cellobiose is known to induce cellulolytic enzymes in the model organism Thermobifida fusca, but only Nocardiopsis dassonvillei showed higher cellulolytic activity in the presence of cellobiose. In T. fusca, cellulases and a putative cellobiose ABC transporter are regulated by the transcriptional regulator CelR. Nine organisms appear to use the CelR site or a closely related binding site to regulate an ABC transporter. In some, CelR also regulates cellulases, while cellulases are controlled by different regulatory sites in three organisms. Mining of genome data for cellulose degradative enzymes followed by experimental verification successfully identified several actinobacteria species which were not previously known to degrade cellulose as cellulolytic organisms.

## Introduction

Aerobic cellulolytic actinobacteria and aerobic fungi have been shown to use a system for cellulose degradation consisting of sets of soluble cellulases and hemicellulases. Most of these independent cellulolytic enzymes contain one or more carbohydrate binding domains. This is in contrast to the system found in many anaerobic bacteria and fungi which consists of multienzyme assemblies attached to the outer surface of the cell, the cellulosomes (reviewed in [1]). Cellulosomes are usually anchored to the surface of the cell through protein-protein interactions and to the carbohydrate substrate through carbohydrate binding domains on the scaffolding protein or on the catalytic enzymes [1].

Previous work on cellulose degradation in actinobacteria has focused on two model organisms, *Thermobifida fusca* and *Cellulomonas fimi* (reviewed in [2]). The system of *T. fusca* is composed of three non-processive endocellulases (E1/Cel9B, E2/Cel6A, E5/Cel5A), which cleave cellulose at random sites along cellulose chains, two exocellulases (E3/Cel6B and E6/Cel48A), which cleave cellobiose units from the ends of cellulose chains in a processive manner, and one processive endocellulase (E4/Cel9A). The latter enzyme combines features of both endo- and exo-type enzymes: it makes an initial endocellulolytic cleavage followed by release of cellotetraose units from the cleaved substrate [2]. Exocellulase E6/Cel48A and processive endocellulase E4/Cel9A remove cellooligosaccharides from the reducing end, while exocellulase E3/Cel6B acts on the nonreducing end [3]. Synergism is observed between exo- and endocellulases (endo/exo synergism) or when different classes of exocellulases are combined (exo/exo synergism); processive endocellulase displays synergism with both exo- and endocellulases. A transcription factor regulating the expression of *T. fusca* cellulases (CelR) has been identified, and in vitro experiments indicate that cellobiose acts as an effector causing dissociation of the CelR-DNA complex [4]. The set of cellulases in *C. fimi* is also comprised of three endocellulases (CenA, CenB and CenD), two exocellulases (CbhA and CbhB), and a processive endocellulase CenC [2]. While these belong to the same glycosyl hydrolase families as the corresponding *T. fusca* enzymes, the sequences are not closely related in most cases.

The Genomic Encyclopedia of Bacteria and Archaea (GEBA) project has generated genome sequences for a number of actinobacteria [5]. Four of these organisms are known to degrade cellulose: *Cellulomonas flavigena* 134, DSM 20109 [6], *Thermobispora* (formerly *Microbispora*) *bispora* R51, DSM 43833 [7], *Thermomonospora curvata* DSM 43183 [8], and *Xylanimonas cellulosilytica* XIL07, DSM 15894 [9]. During analysis of the other actinobacterial genomes, we observed that many contained glycosyl hydrolases similar to endo- and exocellulases of *T. fusca* and *C. fimi*, although the organisms were not known to be cellulolytic. These organisms are *Actinospica robiniae* GE134769, DSM 44927, *Actinosynnema mirum* 101, DSM 43827, *Catenulispora acidiphila* ID139908, DSM 44928, *Jonesia denitrificans* 55134, DSM 20603, *Nocardiopsis dassonvillei* IMRU 509, DSM 43111, *Stackebrandtia nassauensis* LLR-40K-21, DSM 44728, and *Streptosporangium roseum* NI 9100, DSM 43021. We present here an analysis of the cellulolytic enzymes of these actinobacteria and comparison with the genomes of known cellulolytic actinobacteria as well as experimental demonstration of cellulose degradation by these bacteria.

## Results

### Cellulose degradation: computational analysis of glycosyl hydrolases

**Figure 1:**
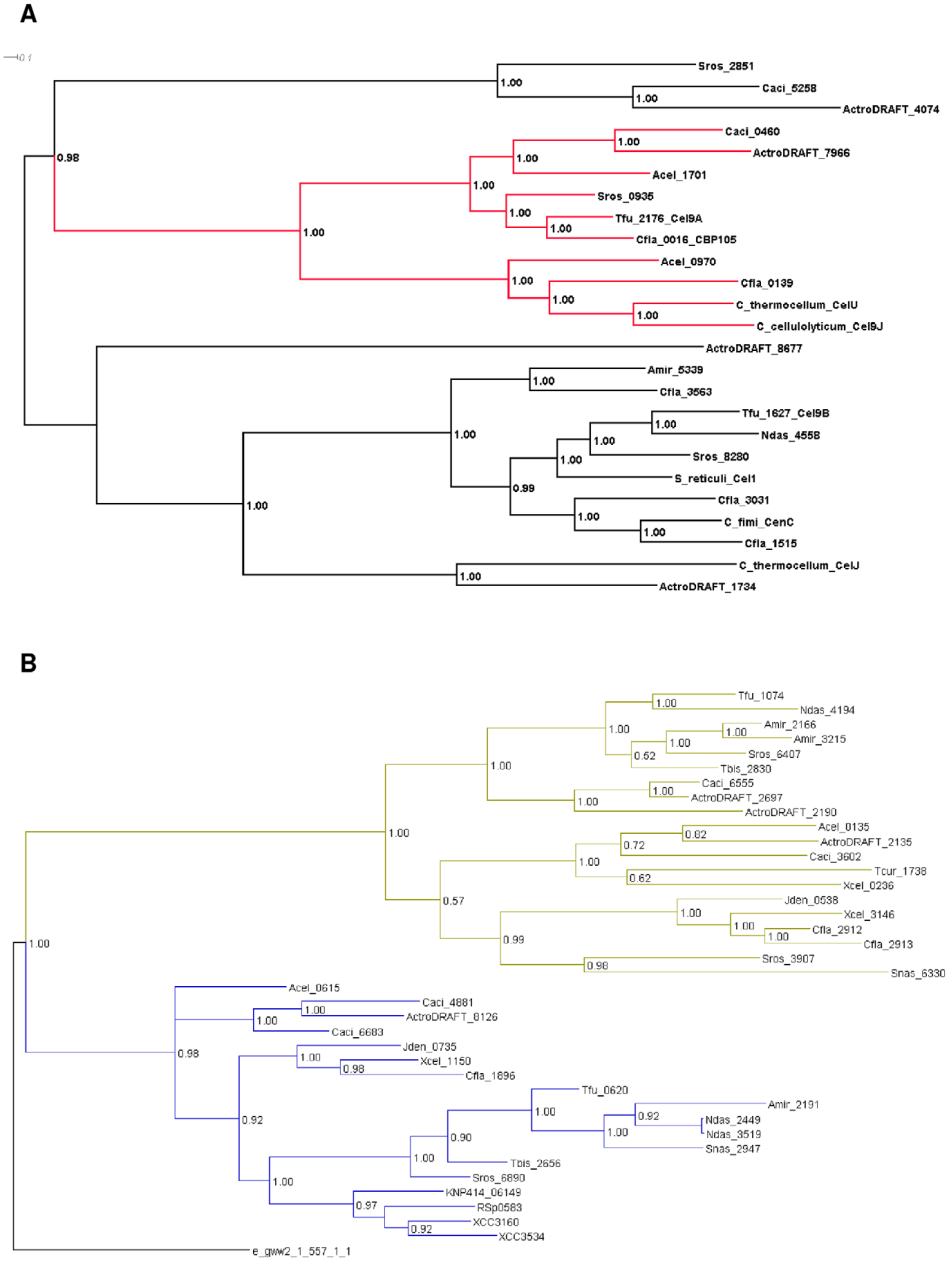
Phylogenetic analysis of family GH9 and GH6 proteins from actinobacterial genomes and experimentally characterized proteins. Only the glycosyl hydrolase domains were included in the alignment. a. GH9 family proteins; branch including “Theme B" proteins is in red. C_cellulolyticum: *Clostridium cellulolyticum*; C_thermocellum: *Clostridium thermocellum*; C_fimi: *Cellulomonas fimi*; S_reticuli: *Streptomyces reticuli*. b. GH6 family proteins; branch including predicted exocellulases is colored blue, branch including predicted endocellulases is colored green. KNP414_06149 – exoglucanase from *Paenibacillus mucilaginosus* KNP414, XCC3160 and XCC3534 – exoglucanases from *Xanthomonas campestris* pv. campestris ATCC 33913; e_gww2_1_557_1_1 – cbhII from *Phanerochaete chrysosporium*; RSp0583 – exoglucanase from *Ralstonia solanacearum* GMI1000.

Experimentally characterized endocellulases are from glycosyl hydrolase families GH5, GH6, GH8, GH9, GH12, and GH51, while predicted exocellulases belong to families GH6 and GH48 according to CAZy classification [10]. While all experimentally characterized actinobacterial GH48 family enzymes are reducing-end exocellulases, GH6 family enzymes may have either endo- or exocellulase activity. Similarly, GH9 family enzymes may have either processive or non-processive endocellulase activity. In order to distinguish between different enzymatic activities within the same GH family we performed phylogenetic analysis of catalytic domains of proteins assigned to GH9 and GH6 families (Figure 1a and 1b, respectively).

GH9 proteins have been assigned to different “themes" based on their sequence similarity and possession of domains other than the glycosyl hydrolase domain [11]. The processive endocellulases found in actinobacteria belong to Theme B, and they are generally composed of a GH9 catalytic domain followed by a carbohydrate-binding CBM3 domain, a fibronectin type 3 (fn3) domain, and a CBM2 or a second CBM3 domain. A processive endocellulase, very similar in sequence to the *T. fusca* enzyme (Tfu_2176, Cel9A), has been identified in *C. flavigena* and is named CBP105 [12]. Four of the other actinobacteria included in the study are likely to have processive endocellulases, based on clustering with the *T. fusca* and *C. flavigena* enzymes in phylogenetic analysis (Figure 1a) and similar domain architecture. Cfla_0139 and Acel_0970 also belong to Theme B, and therefore may be processive endocellulases. If they are, then *C. flavigena* and *A. cellulolyticus* would each have two processive endocellulases. Catalytic domains of GH6 family proteins can be divided into exocellulase subfamily clustering with Tfu_0620 (E3/Cel6B) and endocellulase subfamily clustering with Tfu_1074 (E2/Cel6A) (Figure 1b).

Table 1 summarizes the distribution of putative endo- and exocellulases in the eleven actinobacterial genomes that we studied, along with the cellulases from the previously published genomes of *T. fusca* and *Acidothermus cellulolyticus*. Most genomes included in this study encode genes for predicted endocellulases and two exocellulases, one acting on the non-reducing end of cellulose polymers (GH6 family enzyme) and the other acting on the reducing end (GH48 family enzyme). Two exceptions are *S. nassauensis*, which lacks a reducing end exocellulase of GH48 family, and *T. curvata*, in which no exocellulases were identified. Two pseudogenes with similarity to the reducing- and non-reducing end exocellulases, Tcur_4566 and Tcur_4570 (Table 1), were found in the latter genome suggesting recent loss of cellulose-degrading capability by this *T. curvata* strain.

**Table 1:** Known and predicted cellulases from actinobacteria.

Organism	Endocellulase	Exocellulase	Processive
A. cellulolyticus	**Acel_0614** (GH5) **Acel_0135** (GH6) Acel_0970 (GH9) **Acel_0615** (GH12) Acel_0619 (GH12)	**Acel_0615** (GH6) **Acel_0617** (GH48)	**Acel_1701** (GH9)
A. robiniae	**Actro_1189** (GH5) **Actro_2709** (GH5) **Actro_8514** (GH5) **Actro_2135** (GH6) Actro_2190 (GH6) Actro_2697 (GH6) Actro_1734 (GH9) Actro_1655 (GH12) **Actro_1922** (GH12) **Actro_1014** (GH51) Actro_3019 (GH51) Actro_6327 (GH51) Actro_7002 (GH51) **Actro_7965** (GH51) Actro_8127 (GH51)	Actro_8126 (GH6) **Actro_0272** (GH48) Actro_7971 (GH48)	**Actro_7966** (GH9)
A. mirum	Amir_2781 (GH5) **Amir_3216** (GH5) Amir_3335 (GH5) **Amir_2166** (GH6) **Amir_3215** (GH6) **Amir_5339** (GH9) Amir_3027 (GH12) Amir_3987 (GH12) **Amir_2048** (GH51)	**Amir_2191** (GH6) **Amir_2167** (GH48)	
C. acidiphila	**Caci_4214** (GH5) **Caci_4876** (GH5) **Caci_4946** (GH5) Caci_5006 (GH5) **Caci_3602** (GH6) **Caci_6555** (GH6) **Caci_3603** (GH12) Caci_4157 (GH51) Caci_4289 (GH51) Caci_6262 (GH51) **Caci_6640** (GH51)	**Caci_4881** (GH6) Caci_6683 (GH6) Caci_3604 (GH48)	**Caci_0460** (GH9)
C. flavigena	Cfla_1897 (GH5) **Cfla_2912** (GH6) **Cfla_2913** (GH6) **Cfla_0139** (GH9) **Cfla_1515** (GH9) Cfla_3031 (GH9) **Cfla_3563** (GH9)	**Cfla_1896** (GH6) **Cfla_3105** (GH48)	**Cfla_0016** (GH9)
J. denitrificans	Jden_0734 (GH5) Jden_0538 (GH6)	Jden_0735 (GH6) Jden_1134 (GH48)	
N. dassonvillei	Ndas_1368 (GH5) **Ndas_4194** (GH6) **Ndas_4558** (GH9)	**Ndas_2449** (GH6) **Ndas_3519** (GH6) **Ndas_2448** (GH48)	
S. nassauensis	Snas_1941 (GH5) **Snas_6330** (GH6)	**Snas_2947** (GH6)	
S. roseum	Sros_3907 (GH6) **Sros_6407** (GH6) Sros_1551 (GH8) **Sros_8280** (GH9)	**Sros_6890** (GH6) **Sros_0936** (GH48)	Sros_0935 (GH9)
T. fusca	**Tfu_0901** (GH5) Tfu_2712 (GH5) **Tfu_1074** (GH6) **Tfu_1627** (GH9)	**Tfu_0620** (GH6) **Tfu_1959** (GH48)	**Tfu_2176** (GH9)
T. bispora	**Tbis_2830** (GH6) Tbis_0352 (GH12)	**Tbis_2656** (GH6) **Tbis_2138** (GH48)	
T. curvata	**Tcur_1738** (GH6)	Tcur_4570 (GH6)* Tcur_4566 (GH48)*	
X. cellulosilytica	Xcel_0182 (GH5) **Xcel_0236** (GH6) Xcel_3146 (GH6)	Xcel_1150 (GH6) Xcel_1153 (GH48)	

* – pseudogenes.

With the exception of GH6-family exocellulase from *S. nassauensis*, all other exocellulases found in actinobacterial genomes included in this study have at least one non-catalytic carbohydrate-binding module (CBM), which play an important role in hydrolysis of insoluble cellulosic substrates [13]. Analysis of exocellulase domain architecture revealed common themes of catalytic and CBM domain arrangement. Reducing end exocellulases of GH48 family can be grouped into 2 types: those with N-terminal CBM (type I or *Thermobifida*-like) and those with C-terminal CBM (type II or *Cellulomonas*-like) (Figure S1a). Similarly non-reducing-end exocellulases of GH6 family can be grouped into *Thermobifida*-like type I with N-terminal CBM and *Cellulomonas*-like type II with C-terminal CBM (Figure S1b). The domain arrangement of non-processive endocellulases of GH6 family appears to be the reverse of that of GH6 family exocellulase with *Thermobifida*-like type I having C-terminal CBM and *Cellulomonas*-like type II having N-terminal CBM (Figure S1c). Considering that both reducing and non-reducing end exocellulases are present in 10 out of 12 genomes included in the study, one would expect to find many combinations of different domain architectures. Instead, a remarkable conservation is observed: those organisms possessing type I reducing-end exocellulase also have type I non-reducing end exocellulase and type I non-processive endocellulase of GH6 family (*Thermobifida* type, Table S1). Likewise, actinobacteria with type II reducing-end exocellulase have type II non-reducing end exocellulase and mostly type II non-processive endocellulase (*Cellulomonas* type, Table S1). We hypothesize that this non-random distribution of enzymes with different domain architectures may reflect optimization of actinobacterial cellulase system to achieve maximal synergy between endo- and exocellulases. Two exceptions from this conserved domain architecture are *C. acidiphila* and *A. robiniae*, which contain enzymes with both types of domain arrangements. Both genomes have the largest and the most diverse sets of glycosyl hydrolases as compared to other actinobacteria included in this study (see below). The catalytic activity and expression of the enzymes in these organisms require further experimental investigation.

### Cellulose degradation: computational analysis of auxiliary genes

In addition to predicted cellulases, we identified genes in these actinobacteria for beta-glucosidases, beta-glucan glucohydrolases and cellobiose phosphorylases, enzymes required for cellobiose utilization within the cell (Table S2). All of the organisms have at least one beta-glucosidase, most have beta-glucan glucohydrolases, and two (*C. flavigena* and *X. cellulosilytica*) have cellobiose phosphorylases. The beta-glucosidases belong to families GH1 and GH3, the beta-glucan glucohydrolases belong to family GH3, and the cellobiose phosphorylases belong to family GH94.

**Figure 2:**
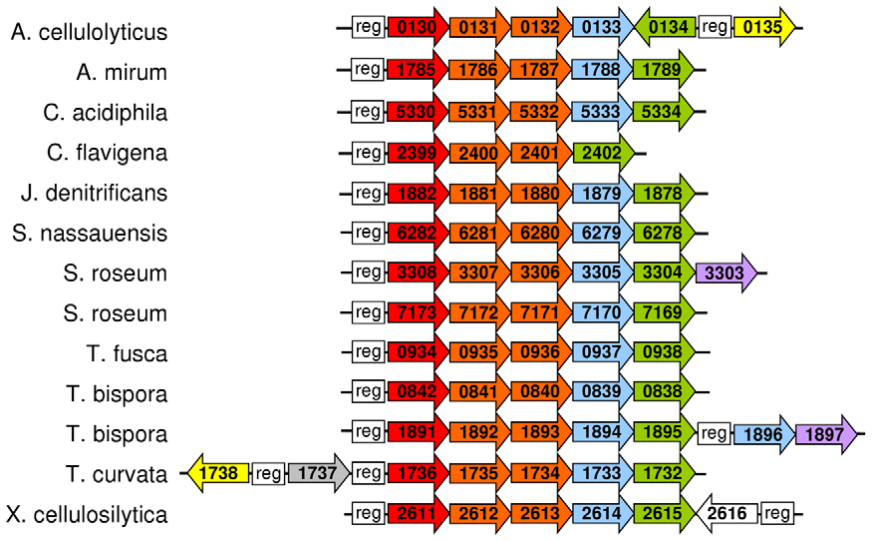
Operons with ABC transporters, beta-glucosidases, and LacI family regulators in actinobacteria. Red: ABC transporter substrate binding protein; orange: ABC transporter membrane protein; grey: ABC transporter ATPase protein; blue: beta-glucosidase; green: LacI family transcriptional regulator; yellow: cellulase; purple: aldose 1-epimerase; white: cellobiose phosphorylase. Reg: regulatory site. Numbers refer to the locus tags of the proteins; for example, 0130 indicates the gene with locus tag Acel_0130.

Another component required for cellulose degradation is a transporter for cellobiose. All of the actinobacteria considered here, except *A. robiniae* and *N. dassonvillei*, have an ABC transporter whose binding protein has at least 48% similarity to that of a characterized cellobiose/cellotriose ABC transporter from *Streptomyces reticuli* [14]. In most cases this ABC transporter is adjacent to a beta-glucosidase and a LacI family transcriptional regulator (Figure 2), although in *C. flavigena* a beta-glucosidase is not present. The beta-glucosidases generally lack signal peptides and thus are predicted to be intracellular, with the possible exception of Xcel_2614, which has a signal peptide probability of 0.465. For most of the ABC transporters, only the substrate binding protein and membrane proteins are found together; however, in *T. curvata* an ABC transporter ATPase protein (Tcur_1737) is adjacent to the other subunits (Figure 2). *S. roseum* and *T. bispora* have two copies of the ABC transporter operon; however, the proteins making up the operons are not closely related and therefore do not appear to result from recent duplications.

### Cellulose degradation: experimental verification

**Figure 3:**
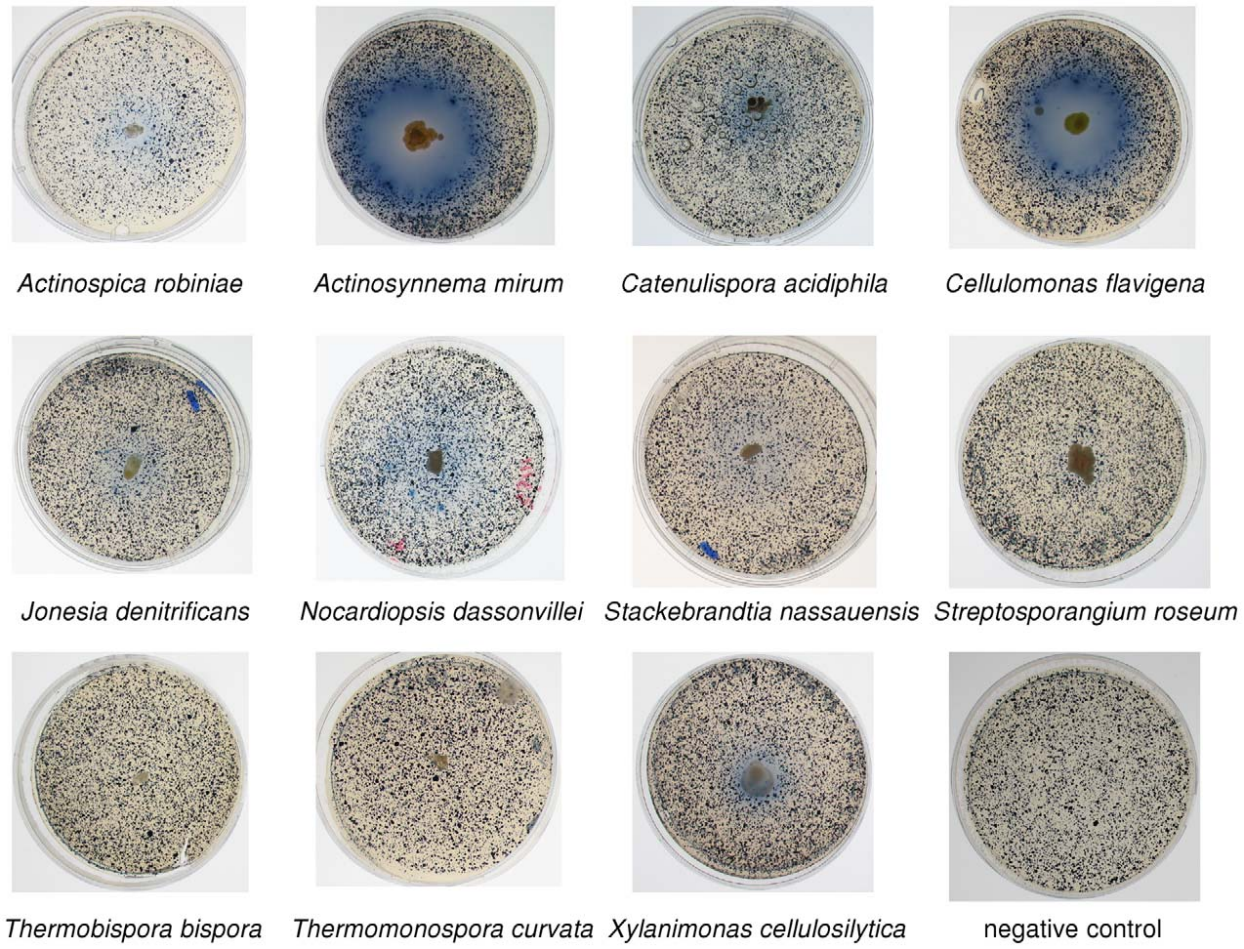
AZCL-HEC assays of eleven actinobacteria. Plates contained 1.5% agar, 0.5% yeast extract, and 0.2% AZCL-HEC. Photographs were taken after seven days. The *N. dassonvillei* plate shown here had 0.01% cellobiose added. The *C. acidiphila* and *A. robiniae* plates were at pH 5.5; others were at pH 7.0.

Based on the above predictions of the presence of cellulases, we performed experiments to determine whether these organisms actually secrete active cellulolytic systems. Table 2 shows the results of several cellulase assays performed on the eleven actinobacteria. In the clearing test, which was performed with quite recalcitrant microcrystalline cellulose, only *A. mirum* gave a positive result, while in the filter paper test, *A. mirum*, *C. flavigena*, and *X. cellulosilytica* showed cellulolytic activity. We also tested these actinobacteria on azurine cross-linked hydroxyethylcellulose (AZCL-HEC) plates, where a blue color indicates cellulolytic activity. *A. mirum* and *C. flavigena* gave the strongest response (Figure 3), and altogether eight of the actinobacteria were positive in this assay (Table 2, Figure 3). *C. acidiphila* gave a positive result on AZCL-HEC plates only when grown at its optimal pH of 5.5. Two of the organisms showed no reproducible cellulolytic activity in AZCL-HEC test: *T. bispora*, and *T. curvata*, and for one organism (*S. roseum*) the results were inconsistent: while in the initial testing it displayed some cellulolytic activity, this observation could not be confirmed in later tests. Since cellobiose is known to induce cellulases in *T. fusca*, we tested whether addition of 0.01% cellobiose would induce cellulolytic activity. *N. dassonvillei* showed a stronger response on AZCL-HEC plates when cellobiose was present (Table 2), but there was no effect on the other organisms.

**Table 2:** Results of cellulase activity assays.

DSM	strain	filterpaper		clearing		AZCL-HEC	
	cellobiose	−	+	−	+	−	+
44927	*Actinospica robiniae*					+	+
43827	*Actinosynnema mirum*	+	+	+	+	++	++
44928	*Catenulispora acidiphila*					+	+
20109	*Cellulomonas flavigena*	+	+			++	++
20603	*Jonesia denitrificans*					+	+
43111	*Nocardiopsis dassonvillei*					(+)	+
44728	*Stackebrandtia nassauensis*					+	+
43021	*Streptosporangium roseum*						
43833	*Thermobispora bispora*						
43183	*Thermomonospora curvata*						
15894	*Xylanimonas cellulosilytica*	+	+			+	+

### Transcriptional regulation

In *T. fusca*, cellulase production is regulated by cellobiose through the LacI family transcriptional regulator CelR [4]. We checked to see if the actinobacteria studied here have homologs of *T. fusca* CelR. A phylogenetic tree was constructed, composed of proteins that have at least 1e^−50^ BLASTp score to *T. fusca* CelR (Figure S2). All of the actinobacteria included in the study have at least one CelR-related transcriptional regulator within the specified cutoff, and some of them have several close homologs of CelR, the highest number being four in *A. robiniae* and *S. roseum*.

A phylogenetic cluster is formed by *T. fusca* CelR, Ndas_0809, Sros_3304, Tbis_1895, Tcur_1732, and Snas_6278. With the exception of Ndas_0809, these transcriptional regulators are found in operons with putative cellobiose ABC transporters (Figure 2). *N. dassonvillei* does not have an ABC transporter similar to the putative cellobiose ABC transporters. The other LacI family transcriptional regulators in operons with putative cellobiose ABC transporters form separate clusters in the tree. Caci_5334, Cfla_2402, Jden_1878, and Xcel_2615 form a cluster, while Tbis_0838 and Sros_7169 are found in another cluster, and Acel_0134 is in a cluster with another protein from the same organism. Amir_1789 is the deepest branch among the actinobacterial CelR homologs included in the phylogenetic analysis, and it is found in an operon with putative cellobiose transporter. Based on the *T. fusca* model, the regulators that are in operons with putative cellobiose ABC transporters are the most likely orthologs of CelR.

In the genome sequence of *T. fusca*, perfect CelR binding sites and also binding sites with one mismatch were found upstream of glycosyl hydrolases [15]. This suggests that in some cases single base changes in the CelR site may still result in a functional binding site; however, it is possible that some sites with single base changes may have reduced affinity for CelR and may not be functional binding sites. The only binding site that has been experimentally tested is the palindromic site from the CelE gene [4]. We searched the actinobacterial genomes for sequences corresponding to perfect CelR binding sites and sites with one mismatch from the palindromic CelR site. The number of sites ranged from zero in *A. cellulolyticus* to 32 in *N. dassonvillei* (end exocellulase and mostly type II non-processive endocellulase (*Cellulomonas* type, Table S3). Genes predicted to be regulated by CelR include cellulases and other glycosyl hydrolases, proteins with chitin or cellulose binding domains, transporters, transcriptional regulators, enzymes involved in carbohydrate metabolism, and signal transduction proteins. Glycosyl hydrolases putatively regulated by CelR or another regulator (see below) are in bold in Table 1 and Table S2.

Five of the organisms had between one and three sites that all had one mismatch from the consensus CelR site, and *A. cellulolyticus* had no CelR sites. Therefore it was doubtful whether CelR was the major regulator of cellulase gene expression in these organisms. We searched for potential cellulase regulatory sites by compiling the upstream 300 nucleotides from the cellulases and beta-glucosidases for each organism and using MEME to search for new sites. *A. cellulolyticus* was found to have a putative regulatory site similar to the CelR binding site, but with one base changed in each half-site of the palindrome: TGGGA(A/T)CG(A/T)TCCCA. Four perfect matches and three single mismatches to this site were found in the genome (Table S3). A site very different from CelR was found in *C. acidiphila*. The site, (G/C)(G/A)(T/A)G(A/G)AA(G/C)TTTC(G/A) is partially palindromic (GAAA(G/C)TTTC) with additional conserved nucleotides on each side of the palindrome. The site was found in 22 copies in the genome (Table S4). In *C. flavigena* two similar potential regulatory sequences were found – CNAA(T/A)CGNTTANNNA and CNAA(T/A)CGNTTCNNNG. These were found 15 times in the genome (Table S4). In *A. robiniae* the sequence TG(A/T)AA(G/T)(C/T)T(G/T)C(A/T) was found in 36 places in the genome (Table S4). Many of the *A. robiniae* sites are palindromic, with the sequence TGAAANTTTCA, similar to the site in *C. acidiphila*. No putative regulatory sites were found in *J. denitrificans* or *X. cellulosilytica* other than CelR sites with one mismatch.

In *T. fusca*, a perfect palindromic CelR site is found upstream of an operon containing a putative cellobiose ABC transporter, a beta-glucosidase, and CelR itself (Figure 2). In *A. mirum*, *J. denitrificans*, *S. nassauensis*, *S. roseum* (Sros_3304–3308), *T. bispora* (Tbis_1891–1895), *T. curvata*, and *X. cellulosilytica*, CelR sites are found upstream of similar operons (Table S3). We checked the remaining operons (Figure 2) to see if CelR or CelR-related sites are found upstream. Upstream of Acel_0130 is an *A. cellulolyticus* regulatory site with two mismatches (TGGGAACGTTCCGC), and upstream of Acel_0134 is a site with one mismatch (TGGGAACGGTCCCA). The similarity of these sites to the sites upstream of glycosyl hydrolases (see above) suggests that Acel_0134 is the regulator for both the ABC transporter operon and the glycosyl hydrolases. Upstream of Caci_5330 are two identical palindromes similar to the CelR site but with one base change in each half-site (TGAGAGCGCTCTCA). The *C. flavigena* ABC transporter operon has a CelR site with two mismatches upstream (TGGGAACGCTCCCG). The upstream region of Sros_7173 has two potential regulatory sites – a CelR site with two mismatches (TGGGAGCGCTCCAT) and a perfect palindrome with two base changes per half site from the CelR site (GGAGAGCGCTCTCC) – suggesting this operon may be regulated by two transcriptional regulators. In the Tbis_0842 upstream region there are three potential regulatory sites – a perfect palindrome identical to the two upstream of Caci_5330, a second perfect palindrome with one base change per half site relative to the first palindrome (AGAGAGCGCTCTCT), and a third site with one base pair changed relative to the second palindrome (AGGGAGCGCTCTCT). Thus all nine of the actinobacteria that have putative cellobiose ABC transporter operons potentially regulate them with CelR or CelR-like transcriptional regulators. The genomes with LacI family regulators that phylogenetically cluster with *T. fusca* CelR (Figure S2) all contain the CelR site.

We checked to see if these palindromes upstream of ABC transporters occur in other places in the genomes (Table S3). The *C. acidiphila* palindrome is found upstream of a second LacI family transcription factor (Caci_6684) and an adjacent glycosyl hydrolase of GH5 family protein with unknown enzymatic activity. It is also found upstream of two GH16 glycosyl hydrolases that may be endo-1,3-beta-glucanases. The *S. roseum* palindrome is found upstream of five genes adjacent to each other on the chromosome (Sros_3721–3725). These proteins include a LacI family transcription regulator, two proteins with CBM32 domains, and a GH3 family protein of unknown function. The *T. bispora* palindromes are only found upstream of the ABC transporter operon. Therefore, in these three organisms, the palindromic sites are found in the vicinity of few additional genes, and these genes do not include cellulase-degrading enzymes.

### Organism details

An analysis of the *A. cellulolyticus* genome has been published [16]. *A. cellulolyticus* has a chromosomal cluster of six glycosyl hydrolases (Acel_0614-Acel_0619) as well as other glycosyl hydrolases scattered within the genome. In the cluster, one of the genes (Acel_0615) has two glycosyl hydrolase domains, GH6 and GH12. We predict that the GH6 domain is an exocellulase while the GH12 domain is an endocellulase (Table 1). Also, based on the GH9 phylogenetic tree (Figure 1a), we predict that Acel_1701 is a processive cellulase. *A. cellulolyticus* appears to have a transcriptional regulatory site similar to the CelR site, and perfect matches to this site are found in four places within the genome – the upstream regions of the glycosyl hydrolase operon and the processive cellulase as well as two hypothetical proteins (Table S3). Two sites with a single mismatch were found upstream of an additional glycosyl hydrolase. In addition this site may regulate a cellobiose ABC transporter operon.

The permanent draft genome of *A. robiniae* shows that this organism has a wide variety of enzymes for cellulase degradation including a processive cellulase (Table 1). Half of the cellulolytic enzymes appear to be coordinately regulated through a site different from CelR. In addition to cellulases, the regulatory site is found upstream of five xylanases belonging to families GH10, GH11, and GH30, and five GH54 arabinofuranosidases (Table S4), suggesting that cellulose and hemicellulose degradation are coregulated. *A. robiniae* does not have an ABC transporter closely related to the *S. reticuli* cellobiose ABC transporter.

*A. mirum* had strong positive reactions in the tests for cellulase production. It appears to use CelR to regulate cellulase production as it has 15 perfect matches to the CelR palindrome and 10 sites with a single base change. Over half of the predicted cellulases including both exocellulases are regulated by CelR. Several other glycosyl hydrolases are likely to be regulated by CelR, including a xyloglucanase, two endoxylanases, a pectate lyase, and a chitinase. Among the other genes regulated by CelR are three genes with the CBM33 carbohydrate binding domain but no glycosyl hydrolase domain. One of these three genes also has a CBM2 domain. Similar proteins are found in *T. fusca*, and they may have a role in making cellulose more accessible for degradation [17]. The putative cellobiose ABC transporter is also regulated by CelR in *A. mirum*.

*C. acidiphila* was not known to be cellulolytic, and was found not to grow on cellulose [18], but it has a large number of predicted cellulases and shows cellulose degradation activity when grown at its optimal pH. *C. acidiphila* does not appear to use the CelR site, but has a similar palindromic site upstream of the putative cellobiose ABC transporter. A separate binding site was identified upstream of about half of the cellulases, an alpha-fucosidase, an alpha-L-arabinofuranosidase, an endoxylanase, and a protein with CBM32 and fn3 domains.

As expected, *C. flavigena* gave strong positive reactions in the filter paper and AZCL-HEC assays. A site unrelated to CelR is found upstream of almost all of the cellulases and also upstream of a GH10-GH62 fusion protein which has predicted xylanase and arabinofuranosidase activities. *C. flavigena* has a probable cellobiose ABC transporter, but uniquely among the actinobacteria in this study it does not have an adjacent beta-glucosidase. The ABC transporter and a GH9 endoglucanase may be regulated by a CelR-related protein.

*J. denitrificans* displayed weak cellulose degradation in the AZCL-HEC assay. Only four potential cellulases were identified in the genome sequence, and, unique among the bacteria considered here, none of the cellulases appear to be regulated by CelR or another regulator. *J. denitrificans* does, however, have a probable cellobiose ABC transporter adjacent to a beta-glucosidase and a LacI family transcriptional regulator, and these may be regulated by CelR.

*N. dassonvillei* showed some cellulase activity on AZCL-HEC plates, and it was the only organism to show increased cellulase activity if cellobiose was added. It has eleven perfect CelR binding sites and 21 single mismatches. *N. dassonvillei* does not have an ABC transporter for cellobiose that is found in most of the other actinobacteria, but it does have an MFS transporter regulated by a perfect CelR site. *N. dassonvillei* has six predicted cellulases, and five of these appear to be regulated by CelR. In addition to cellulases, a rhamnogalacturonan lyase also may be regulated by CelR. Some additional glycosyl hydrolases not involved in plant cell wall degradation also may be regulated by CelR, including a maltodextrin glucosidase and an endo-beta-1,3-glucanase.

*S. nassauensis* had a weak positive result for cellulase activity on AZCL-HEC plates. Two endocellulases and one exocellulase were found in the genome. It appears to use the CelR site for regulation, as three perfect matches and six single mismatches were found. These sites regulate one exocellulase and one endocellulase, a carbohydrate binding protein (CBM33 domain), a beta-glucosidase, and a cellobiose ABC transporter, as well as several hypothetical proteins.

In the *S. roseum* genome, four endocellulases, two exocellulases, and a processive cellulase were found. CelR may be involved in cellulose regulation as there were eight perfect matches and seven single mismatches to the CelR site. These were found in the vicinity of several cellulases, a beta-glucosidase, a beta-glucan glucohydrolase and a probable cellobiose ABC transporter. In addition sites were found close to genes related to pyruvate dehydrogenase and a protein kinase. *S. roseum* displayed cellulase activity on AZCL-HEC plates once, but this could not be repeated, so it was marked as negative in all cellulase assays (Table 2).

Two exocellulases and two endocellulases were found in the *T. bispora* genome. This genome also had nine perfect matches to the CelR regulatory site, found close to cellulases, beta-glucosidases, a cellobiose ABC transporter and two protein kinases; however, our assays did not detect cellulase activity in this organism.

Only one cellulase was found in the *T. curvata* genome. This cellulase is close to a perfect CelR site. Another perfect CelR site was found next to a transcriptional regulator, a beta-glucosidase, and the components of a cellobiose ABC transporter. No cellulase activity was detected for this organism. The strain of *T. curvata* that showed cellulase activity was found to actually be a strain of *T. fusca* [19]. An endoglucanase was purified from this organism and its N-terminal amino acid sequence was determined to be DEVDEIRNGDFS [20]. This sequence does not match any genes in the *T. curvata* genome, but it has a close hit to the sequence directly after the predicted signal peptide of the *T. fusca* Tfu_1627 (E1, Cel9B) gene (DEVNQIRNGDFS).

*X. cellulosilytica* was one of only three of the tested actinobacteria to show cellulolytic activity in the filter paper assay, and it was also positive in the AZCL-HEC assay. Five cellulases were identified in the genome, and *X. cellulosilytica* is one of the two actinobacteria to have a cellobiose phosphorylase. Three single mismatches were found to the CelR regulatory site, all involved in regulation of enzymes and a transporter involved in cellulase degradation. No additional regulatory site for cellulases was found in this organism.

## Discussion

In the two most studied model organisms for cellulose utilization in actinobacteria, *T. fusca* and *C. fimi*, six cellulases have been identified, belonging to families GH5, GH6, GH9, and GH48; *S. coelicolor* has genes related to five of the six *T. fusca* cellulases [2]. In this study we examined the cellulolytic potential of eleven diverse actinobacteria for which the genome sequences have recently been determined. Eight out of eleven strains used in this study demonstrated reproducible cellulase activity in AZCL-HEC test, and three of them also showed activity on filter paper. We found putative cellulases from the same families as *T. fusca*, but there were also numerous cellulases from families GH12 and GH51, and one from GH8, showing that there is more diversity in actinobacterial cellulases than previously known. In addition, non-random distribution of exo- and endocellulases with conserved domain architectures has been found in these newly identified cellulose degraders. Some of the newly sequenced actinobacterial genomes contain much higher numbers of cellulases than *T. fusca*. For example, *C. acidiphila* and *A. robiniae* have 15 and 19 predicted cellulases, respectively. The cellulases identified here may be useful in the production of biofuels from lignocellulosic materials.

The paradigm for cellulose degradation in actinomycetes involves cellulose degradation to cellobiose outside the cell by uncomplexed enzymes, cellobiose transport into the cell by an ABC transporter [14], and intracellular hydrolysis to form glucose [21]. Analysis of the genomes shows that this process seems to be largely conserved in the other actinobacteria. Almost all have an ABC transporter similar to the characterized *S. reticuli* cellobiose transporter, and adjacent to the transporter in most genomes is an intracellular beta-glucosidase (Figure 2). Only *A. robiniae* and *N. dassonvillei* lack putative cellobiose ABC transporters. Two of the actinobacteria have cellobiose phosphorylase, which gives an advantage to anaerobic cellulolytic organisms [22]. *C. flavigena* can grow by respiration or by fermentation [23], so cellobiose phosphorylase may be important under fermentative conditions. The other organism that has a cellobiose phosphorylase is *X. cellulosilytica*, and it is currently unknown whether this organism grows fermentatively.

We also found some diversity in transcriptional regulation of cellulose degradation. The CelR regulatory site or a related palindrome appears to be used by ten of the eleven organisms studied here and is always found to regulate a putative cellobiose ABC transporter, with the exception of *N. dassonvillei*, which does not have this ABC transporter. In many of the organisms the CelR site is also used to regulate cellulose degradative enzymes, similar to the situation in *T. fusca*. However, in three of the organisms a site very different from CelR was found upstream of cellulases and hemicellulases, showing that regulation of cellulose degradation and regulation of cellobiose uptake are under the control of different regulators and potentially may respond to different inducers.

A new finding is that some regulated genes include signal transduction proteins, particularly protein kinases and transcriptional regulators. Amir_1390 from *A. mirum* and Tbis_2744 from *T. bispora* have weak similarity to the ATPase domain of histidine kinases (pfam02518), while Sros_0943 from *S. roseum* and Tbis_0860 from *T. bispora* have strong similarity to Ser/Thr protein kinases (pfam00069). These proteins share 50% amino acid identity and are both found adjacent to a LacI family transcriptional regulator. In addition to protein kinases, several of the actinobacteria have more than one transcriptional regulator regulated by CelR: two in *C. flavigena*, *S. roseum* and *T. bispora*; three in *A. robiniae*, *T. curvata*, and *C. acidiphila*. These findings suggest that the presence of cellulose or cellobiose affects aspects of actinobacterial physiology in addition to the regulation of cellulolytic enzymes. One potential target for regulation is morphological development. In agreement with this proposal, cellulose degradation in *Streptomyces griseus* was found to be linked to morphological development through a transcriptional regulator. Mutation of the transcriptional repressor of cellobiose metabolism CebR resulted in formation of few aerial hyphae, suggesting that the presence of cellobiose inhibits aerial hyphae and spore formation [24]. Almost all of the actinomycetes included in this study produce aerial hyphae with spores, and it is possible that the presence of cellobiose inhibits this developmental process. Another potential target for regulation is the production of secondary metabolites. Interestingly in *A. mirum* a CelR site is found in the coding region of a polyketide synthase (Amir_4019) and thus may be involved in its regulation.

Two of the organisms studied here did not show cellulase activity in any of the assays despite previous reports of cellulase activity. Four cellulases were identified in the *T. bispora* genome, and three of them appear to be regulated by CelR (Table S3). *T. bispora* has been reported to grow in minimal medium with cellulose, and produces a zone of clearing around the colonies [7]; however, we did not find any evidence of cellulolytic activity. It is possible that *T. bispora* can grow on cellulose but its cellulases do not work with AZCL-HEC, and so we saw no results. The only other major difference between our conditions and those from the previous study is that we did not use a humidity controlled incubator, and the plates gradually dried during the experiment.

The other organism that has been reported to grow on cellulose, but did not show cellulase activity in this study is *T. curvata*; however, the strain studied was found to actually be *T. fusca* (see Results section), and there are no published results showing that other strains of *T. curvata* can degrade cellulose. In the genome of *T. curvata* there is one endocellulase but only pseudogenes with similarity to exocellulases. The lack of exocellulases suggests that this organism indeed is incapable of cellulose degradation. The presence of a cellobiose ABC transporter, a beta-glucosidase, and an endoglucanase regulated by CelR binding sites, as well as exocellulase pseudogenes may indicate that this organism once possessed the ability to utilize cellulose, but subsequently lost this ability.

*S. roseum* also did not reproducibly exhibit cellulolytic activity despite having predicted endocellulases, exocellulases, and a processive cellulase, some of which appear to be regulated by CelR. As suggested for *T. bispora*, perhaps the modified cellulose AZCL-HEC could not be recognized by the *S. roseum* enzymes, or the medium may not have been optimal for cellulase production.

In conclusion, we showed that searching for cellulolytic enzymes in complete genome sequences can successfully identify cellulolytic organisms that previously were not known to be cellulolytic. Of the seven organisms we tested that were not previously known to degrade cellulose, six showed activity in assays for cellulases.

## Materials and Methods

Genome sequencing and automatic annotation have been described for Actinosynnema mirum [25], Catenulispora acidiphila [26], Cellulomonas flavigena [27], Jonesia denitrificans [28], Nocardiopsis dassonvillei [29], Stackebrandtia nassauensis [30], Streptosporangium roseum [31], Thermobispora bispora [32], Thermomonospora curvata [33], and Xylanimonas cellulosilytica [34]. Their genome sequences are available from GenBank. The permanent draft genome of Actinospica robiniae is available in IMG-ER (http://img.jgi.doe.gov/er) [35] and IMG-GEBA (http://img.jgi.doe.gov/geba). Analysis of the genomes was carried out with IMG-ER. Signal peptide analysis was carried out with SignalP [36].

Glycosyl hydrolase coding genes belonging to families that are known to include cellulose-degrading enzymes were identified using Pfam and COG domains. Assignment of function was based on phylogenetic analysis and/or similarity to enzymes of known function. GH6 and GH9 amino acid sequences were aligned with Clustal W [37]. Trees were generated with MrBayes version 3.1.2 [38] using the mixed model with 1,000,000 generations sampled every 100 generations. The first 25% of generations were discarded as burn-in. Trees were displayed with Dendroscope [39].

MEME (http://meme.sdsc.edu) [40] was used to identify potential regulatory sites in the organisms that had few CelR sites. The 300 base pairs upstream of predicted cellulases and beta-glucosidases were compiled. If a glycosyl hydrolase appeared to be part of an operon (genes separated by less than 100 bp), the DNA sequence at the beginning of the operon was used. Motif distribution was set for zero or one motif per sequence, with a motif width of between 6 and 20 nucleotides.

To screen for total cellulase activity, the ability to hydrolyse filter paper was tested. A piece of Whatman paper No1 (2.0×7.0 cm) was put into a 100 ml Erlenmeyer flask filled with 30 ml medium (containing 0.1% yeast extract). About a third of the filter paper stripe was dunked in the medium. Inoculation was carried out with three overgrown agar plugs (0.5×0.5×0.5 cm). The cultures were incubated at the organism's optimal growth temperature without shaking. In case of cellulolytic activity, after 2–4 weeks, the dunked part of the paper stripe is partly solubilised.

The clearing test for total cellulase activity (beta-1,4-endoglucanase and cellobiohydrolase activity) was carried out with microcrystalline cellulose as substrate. The medium contained 0.1% yeast extract and 1.0% cellulose (PF 30, Jelucel; particle size diameter less than 30 µm). After autoclaving, the medium in the tubes was gently shaken while cooling down to avoid sedimentation of the microcrystalline cellulose. About 5–7 weeks after inoculation, activity becomes visible by the clearing of the turbid medium.

Screening for beta-1,4-endoglucanase activity was carried out using agar plates containing hydroxyethylcellulose with a coupled dye. Azurine crosslinked hydroxyethylcellulose (AZCL-HEC) is an insoluble substrate. Cellulolytic activity leads to a release of soluble dye-labelled fragments and this becomes observable by the coloration of the medium around the inoculum. In later stages the insoluble substrate is completely dissolved. Agar plates were prepared with 0.2% AZCL-HEC (Megazyme, Ireland), 0.5% yeast extract, and 1.5% agar. To achieve an even spreading of AZCL-HEC across the petri dish and to avoid its sedimentation, a thin layer of medium containing AZCL-HEC was poured above a layer of dye-free agar containing 0.5% yeast extract.

To test the influence of cellobiose, all three assays were carried out without and with addition of 0.01% cellobiose. In case of the strains *C. acidiphila* and *A. robiniae*, the pH of the medium was adjusted to pH 5.5 with HCl.

## Supporting Information

Figure S1 **Domain architecture of exocellulases and GH6 family non-processive endocellulase found in actinobacterial genomes.** Domains were identified by hmmsearch against the corresponding Pfam models.
(PDF)

Figure S2 **Phylogenetic analysis of CelR and related proteins.** Analysis was carried out with MrBayes 3.1.2 as described in Materials and Methods. Proteins with e^−50^ or lower to *T. fusca* CelR were included in the analysis. LacI family proteins from *Escherichia coli*, *Klebsiella pneumoniae*, and *Serratia proteamaculans* were used as the outgroup.
(PDF)

Table S1 Distribution of exocellulases and GH6 family endocellulases with different domain architectures among actinobacteria included in the study. Organisms with *Thermobifida*-type system are highlighted in yellow, organisms with *Cellulomonas*-type system are highlighted in green. * – this protein has 2 CBMs, N-terminal CBM3 and C-terminal CBM2.
(DOC)

Table S2 Predicted beta-glucosidases, beta-glucan glucohydrolases, and cellobiose phosphorylases in actinobacterial genomes. Locus tags in bold type indicate genes predicted to be under the regulation of a transcription factor. For *A. robiniae*, the word DRAFT was removed from the locus tags. For example, Actro_0265 refers to the locus tag ActroDRAFT_0265.
(DOC)

Table S3 Predicted CelR and related binding sites in actinobacterial genomes. Sites with zero or one mismatches are listed. For *A. robiniae*, the word DRAFT was removed from the locus tag. For example, Actro_0742 refers to the locus tag ActroDRAFT_0742.
(DOC)

Table S4 Predicted non-CelR regulatory sites in *C. acidiphila*, *C. flavigena*, and *A. robiniae*. For *A. robiniae*, the word DRAFT was removed from the locus tag. For example, Actro_0272 refers to the locus tag ActroDRAFT_0272. For DNA regulatory site sequences, please refer to the text.
(DOC)
