# Supplementary material for: Genomics of Aerobic Cellulose Utilization Systems in Actinobacteria
Source: PLoS One. 2012 Jun 18;7(6):e39331. doi: 10.1371/journal.pone.0039331 (PMC3377646; doi:10.1371/journal.pone.0039331)
Supplement: Figure S1 — Domain architecture of exocellulases and GH6 family non-processive endocellulase found in actinobacterial genomes. Domains were identified by hmmsearch against the corresponding Pfam models. (PDF) [file pone.0039331.s001.pdf]

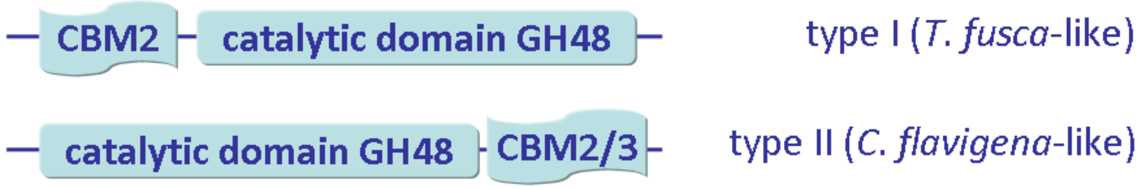

(a) Reducing end exocellulases (GH48)

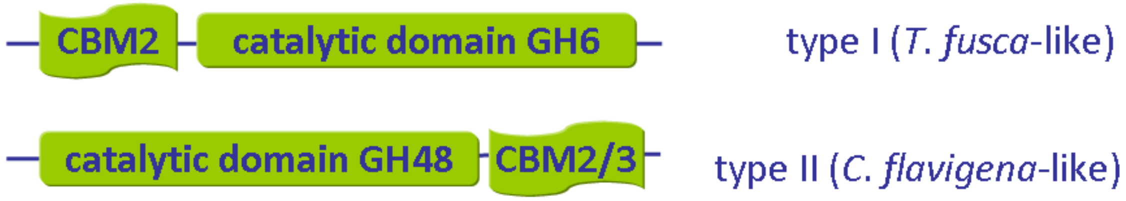

(b) Non-reducing end exocellulases (GH6)

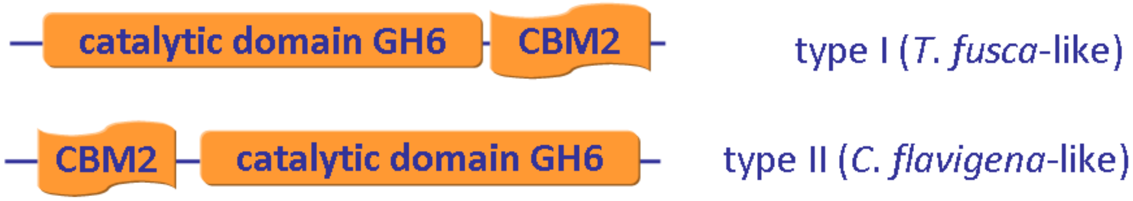

(c) Non-processive endocellulases (GH6)
