# Supplementary figures and images for: Genomics of Aerobic Cellulose Utilization Systems in Actinobacteria
Source: PLoS One. 2012 Jun 18;7(6):e39331. doi: 10.1371/journal.pone.0039331 (PMC3377646; doi:10.1371/journal.pone.0039331)

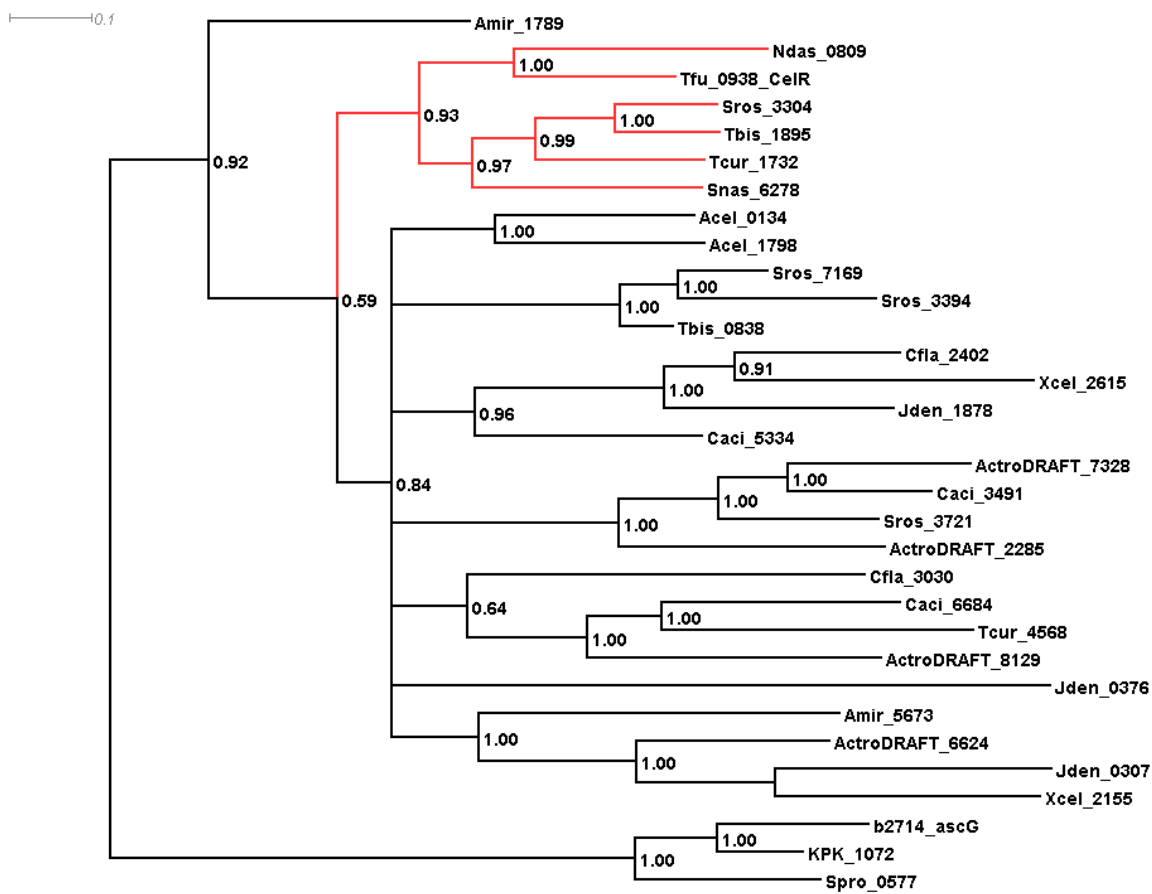

Supplement: Figure S2 — Phylogenetic analysis of CelR and related proteins. Analysis was carried out with MrBayes 3.1.2 as described in Materials and Methods. Proteins with e−50 or lower to T. fusca CelR were included in the analysis. LacI family proteins from Escherichia coli, Klebsiella pneumoniae, and Serratia proteamaculans were used as the outgroup. (PDF) [file pone.0039331.s002.pdf]
