# Supplementary material for: Genomics of Aerobic Cellulose Utilization Systems in Actinobacteria
Source: PLoS One. 2012 Jun 18;7(6):e39331. doi: 10.1371/journal.pone.0039331 (PMC3377646; doi:10.1371/journal.pone.0039331)
Supplement: Table S1 — Distribution of exocellulases and GH6 family endocellulases with different domain architectures among actinobacteria included in the study. Organisms with Thermobifida-type system are highlighted in yellow, organisms with Cellulomonas-type system are highlighted in green. * – this protein has 2 CBMs, N-terminal CBM3 and C-terminal CBM2. (DOC) [file pone.0039331.s003.doc]

|  | Reducing-end exocellulase (GH48) | | Non-reducing end exocellulase (GH6) | | Non-processive endocellulase (GH6) | |
| --- | --- | --- | --- | --- | --- | --- |
|  | Ex I (CBM-GH) | Ex II (GH-CBM) | Ex I (CBM-GH) | Ex II (GH-CBM) | En I (GH-CBM) | En II (CBM-GH) |
| *T. fusca* | Tfu_1959 |  | Tfu_0620 |  | Tfu_1074 |  |
| *A. mirum* | Amir_2167 |  | Amir_2191 |  | Amir_2166, Amir_3215 |  |
| *N. dassonvillei* | Ndas_2448 |  | Ndas_2449, Ndas_3519 |  | Ndas_4194 |  |
| *S. roseum* | Sros_0936 |  | Sros_6890 |  | Sros_6407 |  |
| *T. bispora* | Tbis_2138 |  | Tbis_2656 |  | Tbis_2830 |  |
| *C. flavigena* |  | Cfla_3105 |  | Cfla_1896 |  | Cfla_2912, Cfla_2913 |
| *X.cellulosilytica* |  | Xcel_1153 |  | Xcel_1150 |  | Xcel_3146 |
| *J. denitrificans* |  | Jden_1134 |  | Jden_0735 |  | Jden_0538 |
| *A. cellulolyticus* |  | Acel_0617* |  | Acel_0615 |  |  |
| *A. robiniae* | ActroDRAFT_7971 | ActroDRAFT_0272 | ActroDRAFT_8126 |  | ActroDRAFT_2697 |  |
| *C. acidiphila* |  | Caci_3604 | Caci_6683 | Caci_4881 | Caci_6555 |  |
