# Supplementary material for: Genomics of Aerobic Cellulose Utilization Systems in Actinobacteria
Source: PLoS One. 2012 Jun 18;7(6):e39331. doi: 10.1371/journal.pone.0039331 (PMC3377646; doi:10.1371/journal.pone.0039331)
Supplement: Table S2 — Predicted beta-glucosidases, beta-glucan glucohydrolases, and cellobiose phosphorylases in actinobacterial genomes. Locus tags in bold type indicate genes predicted to be under the regulation of a transcription factor. For A. robiniae, the word DRAFT was removed from the locus tags. For example, Actro_0265 refers to the locus tag ActroDRAFT_0265. (DOC) [file pone.0039331.s004.doc]

Table S2. Glucosidases and cellobiose phosphorylases.

| Organism | Beta-glucosidase/beta-glycosidase | Beta-glucan glucohydrolase | Cellobiose phosphorylase |
| --- | --- | --- | --- |
| A. cellulolyticus | **Acel_0133** (GH1) |  |  |
| A. robiniae | Actro_1612 (GH1)  Actro_3268 (GH1)  Actro_5827 (GH1)  Actro_7284 (GH1)  Actro_1868 (GH3)  Actro_2286 (GH3)  Actro_2776 (GH3)  Actro_2921 (GH3)  Actro_4062 (GH3) | **Actro_6724** (GH3) |  |
| A. mirum | Amir_0033 (GH1)  Amir_1683 (GH1)  **Amir_1788** (GH1)  Amir_2978 (GH3)  Amir_4872 (GH3) | Amir_2168 (GH3)  **Amir_4963** (GH3) |  |
| C. acidiphila | Caci_1349 (GH1)  Caci_2517 (GH1)  **Caci_5333** (GH1)  Caci_1609 (GH3)  Caci_3493 (GH3)  Caci_3845 (GH3)  Caci_4099 (GH3)  Caci_5042 (GH3)  Caci_5444 (GH3)  Caci_6130 (GH3)  Caci_6878 (GH3) | Caci_4677 (GH3)  Caci_4940 (GH3) |  |
| C. flavigena | Cfla_1084 (GH1)  Cfla_3027 (GH3) |  | Cfla_0148 (GH94) |
| J. denitrificans | Jden_1754 (GH1)  **Jden_1879** (GH1)  **Jden_0198** (GH3)  Jden_0471 (GH3)  Jden_2534 (GH3) | Jden_1770 (GH3) |  |
| N. dassonvillei | **Ndas_0808** (GH1)  **Ndas_2534** (GH1)  Ndas_3225 (GH1)  Ndas_1022 (GH3) | Ndas_5566 (GH3) |  |
| S. nassauensis | Snas_4280 (GH1)  **Snas_6279** (GH1)  Snas_2625 (GH3)  Snas_5561 (GH3) |  |  |
| S. roseum | **Sros_3305** (GH1)  Sros_6572 (GH1)  Sros_7167 (GH1)  **Sros_7170** (GH1)  Sros_7951 (GH1)  Sros_6143 (GH3) | **Sros_1295** (GH3)  Sros_6485 (GH3) |  |
| T. fusca | **Tfu_0937** (GH1)  Tfu_1629 (GH1) | Tfu_1607 (GH3) |  |
| T. bispora | **Tbis_0839** (GH1)  **Tbis_1894** (GH1)  **Tbis_1896** (GH1)  **Tbis_2605** (GH1)  Tbis_2763 (GH1) | Tbis_2406 (GH3)  Tbis_2412 (GH3) |  |
| T. curvata | **Tcur_1733** (GH1)  Tcur_2184 (GH1)  Tcur_4773 (GH1) |  |  |
| X. cellulosilytica | Xcel_2440 (GH1)  **Xcel_2614** (GH1)  Xcel_0928 (GH3) | Xcel_1195 (GH3) | **Xcel_2616** (GH94) |
