# Supplementary material for: Genomics of Aerobic Cellulose Utilization Systems in Actinobacteria
Source: PLoS One. 2012 Jun 18;7(6):e39331. doi: 10.1371/journal.pone.0039331 (PMC3377646; doi:10.1371/journal.pone.0039331)
Supplement: Table S3 — Predicted CelR and related binding sites in actinobacterial genomes. Sites with zero or one mismatches are listed. For A. robiniae, the word DRAFT was removed from the locus tag. For example, Actro_0742 refers to the locus tag ActroDRAFT_0742. (DOC) [file pone.0039331.s005.doc]

Table S3. Predicted CelR and related binding sites.

| Position on chromosome | Mismatches | Locus tag of regulated gene | Location of binding site | Gene function |
| --- | --- | --- | --- | --- |
| Acidothermus cellulolyticus – CelR – none | | | | |
| Acidothermus cellulolyticus – TGGGA(A/T)CG(A/T)TCCCA | | | | |
| 652791 | 0 | Acel_0613 | upstream | hypothetical protein |
| 653110 | 0 | Acel_0614 | upstream | GH5 endocellulase |
|  |  | Acel_0615 | upstream | GH6 exocellulase/GH12 endocellulase |
|  |  | Acel_0616 | upstream | GH5 mannanase |
|  |  | Acel_0617 | upstream | GH48 exocellulase |
| 1726093 | 0 | Acel_1529 | upstream | hypothetical protein |
| 1916172 | 0 | Acel_1701 | upstream | GH9 processive cellulase |
| 138960 | 1 | Acel_0134 | upstream | transcriptional regulator, LacI family |
|  |  | Acel_0135 | upstream | GH6 endocellulase |
| 139096 | 1 | Acel_0135 | coding region | GH6 endocellulase |
| 2019303 | 1 | Acel_1777 | coding region | sulfur transfer protein |
| Actinospica robiniae – CelR | | | | |
| 7470558 | 1 | Actro_6619 | upstream | GH53 endogalactanase |
| 7470699 | 1 | Actro_6619 | coding region | GH53 endogalactanase |
| Actinosynnema mirum – CelR | | | | |
| 1961193 | 0 | Amir_1785-1787 | upstream | sugar ABC transporter |
|  |  | Amir_1788 | upstream | GH1 beta-glucosidase |
|  |  | Amir_1789 | upstream | transcriptional regulator, LacI family |
| 1967338 | 0 | Amir_1791 | upstream | GH74 xyloglucanase |
| 2003055 | 0 | Amir_1822 | upstream | CBM2 and CBM33 domain protein |
| 2273094 | 0 | Amir_2048 | upstream | GH51 endoglucanase |
| 2391437 | 0 | Amir_2166 | upstream | GH6 endoglucanase |
| 2391994 | 0 | Amir_2167 | upstream | GH48 exoglucanase |
| 2422348 | 0 | Amir_2191 | upstream | GH6 exoglucanase |
| 2746663 | 0 | Amir_2475 | upstream | CBM33 domain protein |
| 3438295 | 0 | Amir_2972 | upstream | xylose isomerase-like protein |
| 3590334 | 0 | Amir_3108 | upstream | lipase/acylhydrolase w/CBM13 domain |
| 3748187 | 0 | Amir_3216 | upstream | GH5 endoglucanase |
| 4109530 | 0 | Amir_3536 | upstream | Cip1-related protein w/CBM2 |
| 5580380 | 0 | Amir_4693 | upstream | CBM33 domain protein |
| 6048914 | 0 | Amir_5072 | upstream | GH10 endoxylanase |
| 6349390 | 0 | Amir_5339 | upstream | GH9 endoglucanase |
| 1533261 | 1 | Amir_1390 | upstream | anti-sigma factor, Ser/Thr kinase |
| 2111432 | 1 | Amir_1900 | upstream | DUF344 domain protein |
|  |  | Amir_1901 | upstream | membrane protein |
| 2422286 | 1 | Amir_2191 | upstream | GH6 exoglucanase |
| 2645684 | 1 | Amir_2384 | coding region | membrane protein DUF894 |
| 3746491 | 1 | Amir_3213 | upstream | GH43 family protein |
|  |  | Amir_3214 | upstream | GH10 endoxylanase |
|  |  | Amir_3215 | upstream | GH6 endoglucanase |
| 4706759 | 1 | Amir_4019 | coding region | polyketide synthase |
| 5922634 | 1 | Amir_4963 | upstream | GH3 beta-glucan glucohydrolase |
| 5925143 | 1 | Amir_4964 | coding region | pectin esterase/pectate lyase |
| 6348309 | 1 | Amir_5336 | upstream | GH19 chitinase |
| 6959145 | 1 | Amir_5915 | coding region | pseudogene, DNA protecting protein |
| Catenulispora acidiphila – CelR | | | | |
| 3207845 | 1 | Caci_2792 | upstream | GH30 endoxylanase |
| 4033970 | 1 | Caci_3610 | upstream | transcriptional regulator, LuxR family |
| Catenulispora acidiphila – TGAGAGCGCTCTCA | | | | |
| 6143995  6144028 | 0  0 | Caci_5330-5332 | upstream | sugar ABC transporter |
|  |  | Caci_5333 | upstream | GH1 beta-glucosidase |
|  |  | Caci_5334 | upstream | transcriptional regulator, LacI family |
| 7703013 | 0 | Caci_6684 | upstream | transcriptional regulator, LacI family |
|  |  | Caci_6685 | upstream | GH5 family protein |
| 161513 | 1 | Caci_0151 | coding region | YcfA family protein |
| 4645840 | 1 | Caci_4113 | upstream | GH16 endo-1,3-beta-glucanase |
| 8421636 | 1 | Caci_7244 | upstream | GH16 endo-1,3-beta-glucanase |
| Cellulomonas flavigena – CelR | | | | |
| 1681821 | 1 | Cfla_1515 | upstream | GH9 endoglucanase |
| Jonesia denitrificans – CelR | | | | |
| 214112 | 1 | Jden_0198 | upstream | GH3 beta-glucosidase |
| 951755 | 1 | Jden_0890 | upstream | hypothetical protein |
| 2062807 | 1 | Jden_1878 | upstream | transcriptional regulator, LacI family |
|  |  | Jden_1879 | upstream | GH1 beta-glucosidase |
|  |  | Jden_1880-1882 | upstream | sugar ABC transporter |
| Nocardiopsis dassonvillei chromosome – CelR | | | | |
| 996916 | 0 | Ndas_0808 | upstream | GH1 beta-glucosidase |
| 2688536 | 0 | Ndas_2245 | upstream | esterase with CBM2 domain |
| 2743275 | 0 | Ndas_2294 | upstream | GH13 maltodextrin glucosidase |
| 2907587 | 0 | Ndas_2449 | upstream | GH6 exoglucanase |
| 3353984 | 0 | Ndas_2808 | coding region | MFS transporter |
| 4226691 | 0 | Ndas_3519 | upstream | GH6 exoglucanase |
| 4389846 | 0 | Ndas_3658 | upstream | pseudogene, mannobiose ABC transporter binding protein |
| 5010053 | 0 | Ndas_4194 | upstream | GH6 endoglucanase |
| 5400791 | 0 | Ndas_4558 | upstream | GH9 endoglucanase |
| 5620226 | 0 | Ndas_4738 | upstream | acetyltransferase |
| 677547 | 1 | Ndas_0546 | upstream | hypothetical protein |
| 783462 | 1 | Ndas_0637 | upstream | glutamate-cysteine ligase |
| 810046 | 1 | - |  |  |
| 1129271 | 1 | Ndas_0923 | upstream | PL11 rhamnogalacturonan lyase, exo-type |
| 1167219 | 1 | Ndas_0950 | upstream | oxidoreductase, aldo-keto reductase family |
| 1640427 | 1 | Ndas_1334 | coding region | short chain dehydrogenase |
| 1743669 | 1 | - |  |  |
| 1788728 | 1 | Ndas_1461 | upstream | protein with CBM13 domain |
| 1947039 | 1 | Ndas_1590 | upstream | GH16 endo-beta-1,3-glucanase |
| 2469005 | 1 | Ndas_2046 | upstream | oxidoreductase, aldo-keto reductase family |
| 2517110 | 1 | Ndas_2096 | coding region | hypothetical protein |
| 2900135 | 1 | Ndas_2446 | coding region | CBM2 and CBM33 domain protein |
| 2905431 | 1 | Ndas_2448 | upstream | GH48 exoglucanase |
| 2907696 | 1 | Ndas_2449 | upstream | GH6 exoglucanase |
| 3023584 | 1 | Ndas_2534 | upstream | GH1 beta-glucosidase |
| 3940830 | 1 | Ndas_3302 | upstream | GH81 family protein |
| 4226000 | 1 | Ndas_3518 | upstream | CBM33 domain protein |
| 5029351 | 1 | - |  |  |
| 5454147 | 1 | Ndas_4598 | coding region | aldose-ketose isomerase |
| 5676524 | 1 | Ndas_4789 | upstream | hypothetical protein |
| Nocardiopsis dassonvillei plasmid – CelR | | | | |
| 138497 | 0 | Ndas_4997 | upstream | probable phosphatase |
| 31709 | 1 | Ndas_4901 | upstream | oxidoreductase |
| Stackebrandtia nassauensis – CelR | | | | |
| 3105868 | 0 | Snas_2947 | upstream | GH6 exoglucanase |
| 3108341 | 0 | Snas_2948 | upstream | CBM33 domain protein |
|  |  | Snas_2949 | upstream | alcohol dehydrogenase |
| 6626794  6626958 | 0  1 | Snas_6278 | upstream | transcriptional regulator, LacI family |
|  |  | Snas_6279 | upstream | GH1 beta-glucosidase |
|  |  | Snas_6280-6282 | upstream | sugar ABC transporter |
| 2819919 | 1 | Snas_2678 | upstream | DUF1023 family protein |
| 2825815 | 1 | Snas_2685 | upstream | hypothetical protein |
| 5442074 | 1 | Snas_5122 | coding region | hypothetical protein |
| 6157087 | 1 | Snas_5795 | upstream | hypothetical protein |
| 6677904 | 1 | Snas_6330 | upstream | GH6 endoglucanase |
| Streptosporangium roseum – CelR | | | | |
| 957906  957790 | 0  1 | Sros_0936 | upstream, coding region | GH48 exoglucanase |
| 968839 | 0 | Sros_0943 | upstream | Ser/Thr protein kinase |
|  |  | Sros_0944 | upstream | transcriptional regulator, LacI family |
| 3604335  3604466 | 0  0 | Sros_3304 | upstream | transcriptional regulator, LacI family |
|  |  | Sros_3305 | upstream | GH1 beta-glucosidase |
|  |  | Sros_3306-3308 | upstream | sugar ABC transporter |
| 7016194 | 0 | Sros_6407 | upstream | GH6 endocellulase |
| 7539516  7539318 | 0  1 | Sros_6843-6845 | upstream | pyruvate/2-oxoglutarate dehydrogenase subunits |
| 7592561 | 0 | Sros_6890 | upstream | GH6 exoglucanase |
| 9137310 | 0 | Sros_8280 | upstream | GH9 endoglucanase |
| 631932 | 1 | Sros_0622 | upstream | acetyltransferase |
| 1337213 | 1 | Sros_1295 | coding region | GH3 beta-glucan glucohydrolase |
| 1581076 | 1 | Sros_1496 | coding region | oxidoreductase |
| 4294705 | 1 | Sros_3855 | coding region | dimethylglycine oxidase |
| 5543010 | 1 | Sros_5016 | upstream | MOSC domain protein |
| Streptosporangium roseum - GGAGAGCGCTCTCC | | | | |
| 4124591 | 0 | Sros_3721 | upstream | transcriptional regulator, LacI family |
| 4127367 | 0 | Sros_3722 | upstream | GH3 family protein |
| 4128952 | 0 | Sros_3723 | upstream | CBM32/DUF1996 protein |
| 4132354 | 0 | Sros_3724 | upstream | CBM32/GH unclassified protein |
| 4132530 | 0 | Sros_3725 | upstream | hypothetical protein |
| 7922479 | 0 | Sros_7169 | upstream | transcriptional regulator, LacI family |
|  |  | Sros_7170 | upstream | GH1 beta-glucosidase |
|  |  | Sros_7171-7173 | upstream | sugar ABC transporter |
| 4230675 | 1 | Sros_3795 | coding region | transcriptional regulator, LuxR family |
| 6433507 | 1 | Sros_5870 | coding region | proteasome subunit |
| 7482194 | 1 | Sros_6792 | coding region | hypothetical protein |
| 8702104 | 1 | Sros_7891 | upstream | GH81 family protein |
| Thermobispora bispora – CelR | | | | |
| 976167 | 0 | - |  |  |
| 978744 | 0 | Tbis_0860 | upstream | Ser/Thr protein kinase |
|  |  | Tbis_0861 | upstream | sugar binding protein |
| 2187614  2187734 | 0  0 | Tbis_1891-1893 | upstream | sugar ABC transporter |
|  |  | Tbis_1894 | upstream | GH1 beta-glucosidase |
|  |  | Tbis_1895 | upstream | transcriptional regulator, LacI family |
| 2193678  2193624 | 0  1 | Tbis_1896 | upstream | GH1 beta-glucosidase |
|  |  | Tbis_1897 | upstream | aldose 1-epimerase |
| 2484128 | 0 | Tbis_2138 | upstream | GH48 exoglucanase |
| 3116150 | 0 | Tbis_2656 | upstream | GH6 exoglucanase |
| 3213505 | 0 | Tbis_2744 | upstream | anti-sigma factor, Ser/Thr kinase |
| 3314500 | 0 | Tbis_2830 | upstream | GH6 endoglucanase |
| 3058489 | 1 | Tbis_2605 | upstream | GH1 beta-glucosidase |
| Thermobispora bispora – TGAGAGCGCTCTCA | | | | |
| 957681 | 0 | Tbis_0838 | upstream | transcriptional regulator, LacI family |
|  |  | Tbis_0839 | upstream | GH1 beta-glucosidase |
|  |  | Tbis_0840-0842 | upstream | sugar ABC transporter |
| Thermobispora bispora – AGAGAGCGCTCTCT | | | | |
| 957583  957856 | 0  1 | Tbis_0838 | upstream | transcriptional regulator, LacI family |
|  |  | Tbis_0839 | upstream | GH1 beta-glucosidase |
|  |  | Tbis_0840-0842 | upstream | sugar ABC transporter |
| 3826144 | 1 | Tbis_3271 | coding region | membrane fusion protein |
| Thermomonospora curvata – CelR | | | | |
| 2031286 | 0 | Tcur_1732 | upstream | transcriptional regulator, LacI family |
|  |  | Tcur_1733 | upstream | GH1 beta-glucosidase |
|  |  | Tcur_1734-1736 | upstream | sugar ABC transporter |
| 2032837 | 0 | Tcur_1737 | upstream | sugar ABC transporter ATPase |
|  |  | Tcur_1738 | upstream | GH6 endocellulase |
| 1633163 | 1 | Tcur_1431 | coding region | endonuclease |
| 3061036 | 1 | Tcur_2625 | upstream | transcriptional regulator, LysR family |
| 5179402 | 1 | Tcur_4568 | upstream | transcriptional regulator, LacI family |
| Xylanimonas cellulosilytica – CelR | | | | |
| 280417 | 1 | Xcel_0236 | upstream | GH6 endoglucanase |
| 2873853 | 1 | Xcel_2611-2613 | upstream | sugar ABC transporter |
|  |  | Xcel_2614 | upstream | GH1 beta-glucosidase |
|  |  | Xcel_2615 | upstream | transcriptional regulator, LacI family |
| 2882648 | 1 | Xcel_2616 | upstream | GH94 cellobiose phosphorylase |
