# Supplementary material for: Genomics of Aerobic Cellulose Utilization Systems in Actinobacteria
Source: PLoS One. 2012 Jun 18;7(6):e39331. doi: 10.1371/journal.pone.0039331 (PMC3377646; doi:10.1371/journal.pone.0039331)
Supplement: Table S4 — Predicted non-CelR regulatory sites in C. acidiphila, C. flavigena, and A. robiniae. For A. robiniae, the word DRAFT was removed from the locus tag. For example, Actro_0272 refers to the locus tag ActroDRAFT_0272. For DNA regulatory site sequences, please refer to the text. (DOC) [file pone.0039331.s006.doc]

Table S4. Predicted non-CelR regulatory sites in *C. acidiphila*, *C. flavigena*, and *A. robiniae*.

| Position on chromosome | Locus tag of regulated gene | Location of binding site | Gene function |
| --- | --- | --- | --- |
| Catenulispora acidiphila | | | |
| 208735 | Caci_0192 | coding region | hypothetical protein |
| 416392 | Caci_0373 | upstream | CBM32 and fn3 domain protein |
| 524528 | Caci_0460 | upstream | GH9 processive cellulase |
|  | Caci_0461 | upstream | transcriptional regulator |
| 550816 | Caci_0483 | upstream | hypothetical protein |
|  | Caci_0482 | upstream | DUF894 family protein |
| 3997891 | Caci_3584 | upstream | condensation domain protein |
|  | Caci_3583 | upstream | LPS biosynthesis protein |
| 4023290 | Caci_3602 | upstream | GH6 endocellulase |
| 4023414 | Caci_3603 | upstream | GH12 endocellulase |
| 4225650 | Caci_3744 | upstream | transcriptional regulator |
| 4778939 | Caci_4214 | upstream | GH5 endocellulase |
| 5548590 | Caci_4875 | upstream | GH29 alpha-fucosidase |
|  | Caci_4876 | upstream | GH5 endocellulase |
| 5559342 | Caci_4881 | upstream | GH6 exocellulase |
| 5560985 | Caci_4882 | upstream | GH54 arabinofuranosidase |
| 5642528 | Caci_4941 | upstream | GH11 endoxylanase |
| 5645411 | Caci_4945 | upstream | DoxX family protein |
| 5648100 | Caci_4946 | upstream | GH5 endoglucanase |
|  | Caci_4947 | upstream | GH29 family protein |
| 5718771 | Caci_4996 | coding region | DUF1680 domain protein |
| 6667764 | Caci_5767 | upstream | DUF377 predicted glycosidase |
| 6884237 | Caci_5964 | coding region | hypothetical protein |
| 7549319 | Caci_6555 | upstream | GH6 endocellulase |
|  | Caci_6556 | upstream | endonuclease/exonuclease/phosphatase |
| 7650043 | Caci_6640 | upstream | GH51 endocellulase |
| 7703097 | Caci_6684 | upstream | transcriptional regulator |
|  | Caci_6685 | upstream | GH5 family protein |
| 9999805 | Caci_8624 | coding region | Rhs family protein |
| Cellulomonas flavigena | | | |
| 21347 | Cfla_0016 | upstream | GH9 processive cellulase |
| 168738 | Cfla_0139 | upstream | GH9 endocellulase |
| 193279 | Cfla_0157 | upstream | hypothetical protein |
| 464938 | Cfla_0438 | coding region | helicase |
| 1681717 | Cfla_1515 | upstream | GH9 endocellulase |
| 2109044 | Cfla_1896 | upstream | GH6 exocellulase |
| 2752389 | Cfla_2460 | coding region | DMT family transporter |
| 2997537 | Cfla_2688 | upstream | UDP-N-acetylmuramate dehydrogenase |
|  | Cfla_2689 | upstream | MFS family transporter |
|  | Cfla_2690 | upstream | MFS family transporter |
|  | Cfla_2691 | upstream | transcriptional regulator |
| 3125470 | Cfla_2811 | upstream | GH5 mannanase |
| 3224417 | Cfla_2912 | upstream | GH6 endocellulase |
| 3226147 | Cfla_2913 | upstream | GH6 endocellulase |
| 3356832 | Cfla_3024 | upstream | GH10 endoxylanase/GH62 arabinofuranosidase |
|  | Cfla_3025 | upstream | transcriptional regulator |
| 3452561 | Cfla_3105 | upstream | GH48 exocellulase |
| 3952811 | Cfla_3563 | upstream | GH9 endocellulase |
| Actinospica robiniae | | | |
| 332500 | Actro_0272 | upstream | GH48 exocellulase |
|  | Actro_0273 | upstream | condensation domain protein |
| 1087015 | Actro_0988 | upstream | methyltransferase |
|  | Actro_0989 | upstream | acyl-CoA synthetase |
| 1112455 | Actro_1013 | upstream | endoribonuclease |
|  | Actro_1014 | upstream | GH51 endocellulase |
| 1246842 | Actro_1132 | upstream | protein with CBM13 domain |
| 1259028 | Actro_1137 | upstream | protein with CBM13 domain |
|  | Actro_1138 | upstream | GH54 arabinofuranosidase |
| 1264878 | Actro_1140 | upstream | GH10 endoxylanase |
|  | Actro_1141 | upstream | GH54 arabinofuranosidase |
| 1276274 | Actro_1151 | upstream | GH10 exoglucanase/xylanase |
| 1326160 | Actro_1189 | upstream | GH5 endocellulase |
| 1338006 | Actro_1195 | upstream | GH54 arabinofuranosidase |
|  | Actro_1196 | upstream | hypothetical protein |
| 1358488 | Actro_1214 | coding region | hypothetical protein |
| 1613801 | Actro_1462 | coding region | transposase fragment |
| 1830339 | Actro_1656 | upstream | GH54 arabinofuranosidase |
|  | Actro_1657 | upstream | GH30 endoxylanase |
| 1836947 | Actro_1660 | upstream | GH10 endoxylanase |
|  | Actro_1661 | upstream | GH54 arabinofuranosidase |
| 1838994 | Actro_1662 | upstream | GH10 endoxylanase |
| 1842199 | Actro_1663 | upstream | LacI family transcriptional regulator |
|  | Actro_1664 | upstream | GH62 arabinofuranosidase |
| 1848986 | Actro_1667 | upstream | GH29 fucosidase |
|  | Actro_1668 | upstream | GH27 alpha-galactosidase |
| 2122786 | Actro_1869 | overlaps stop codon | alkylmercury lyase |
|  | Actro_1870 | upstream | hypothetical protein |
| 2179658 | Actro_1921 | upstream | ankyrin repeat protein |
|  | Actro_1922 | upstream | GH12 endocellulase |
| 2233198 | Actro_1977 | upstream | GH53 arabinogalactan beta-galactosidase |
| 2395302 | Actro_2135 | upstream | GH6 endocellulase |
|  | Actro_2136 | upstream | low temperature requirement A protein |
| 2661337 | Actro_2379 | upstream | GH11 endoxylanase |
| 3023649 | Actro_2708 | upstream | uncharacterized conserved protein |
|  | Actro_2709 | upstream | GH5 endocellulase |
| 3896324 | Actro_3423 | coding region | GH28 exopolygalacturonase |
| 4249805 | Actro_3742 | upstream | FMN-dependent enzyme |
| 4387634 | Actro_3854 | upstream | protein with CBM2 domain |
| 4491575 | Actro_3934 | coding region | peptidase |
| 5532436 | Actro_4834 | upstream | cytochrome P450 |
|  | Actro_4835 | overlaps stop codon | TatA/E family protein |
| 5556837 | Actro_4852 | upstream | major facilitator superfamily protein |
| 7256027 | Actro_6421 | coding region | TIM barrel protein |
| 7589804 | Actro_6724 | coding region | GH3 beta-glucan glucohydrolase |
| 7768528 | Actro_6886 | upstream | hypothetical protein |
| 9015514 | Actro_7965 | upstream | GH51 endocellulase |
|  | Actro_7966 | upstream | GH9 processive cellulase |
| 9462449 | Actro_8340 | upstream | cold shock protein |
|  | Actro_8341 | upstream | GH92 family protein |
| 9690626 | Actro_8514 | upstream | GH5 endocellulase |
|  | Actro_8515 | upstream | esterase |
| 9703498 | Actro_8528 | upstream | esterase with CBM2 domain |
|  | Actro_8529 | upstream | LacI family transcriptional regulator |
| 9804906 | Actro_8621 | upstream | hypothetical protein with signal peptide |
|  | Actro_8622 | upstream | MarR family transcriptional regulator |
